# Expression profile analysis identifies key genes as prognostic markers for metastasis of osteosarcoma

**DOI:** 10.1186/s12935-020-01179-x

**Published:** 2020-03-30

**Authors:** Xiaoqing Guan, Zhiyuan Guan, Chunli Song

**Affiliations:** 1 Center for Cancer Bioinformatics, Key Laboratory of Carcinogenesis and Translational Research (Ministry of Education), Peking University Cancer Hospital & Institute, Beijing, China; 2 Department of Orthopaedics, Peking University Third Hospital, Beijing, China; 3 Beijing Key Laboratory of Spinal Diseases, 49 North Garden Rd Haidian District, Beijing, China

**Keywords:** Biomarker, Osteosarcoma, Metastasis, Molecular classifier, Prognosis, Gene expression

## Abstract

**Background:**

OS is the most common malignant tumor of bone which was featured with osteoid or immature bone produced by the malignant cells, and biomarkers are urgently needed to identify patients with this aggressive disease.

**Methods:**

We downloaded gene expression profiles from GEO and TARGET datasets for OS, respectively, and performed WGCNA to identify the key module. Whereafter, functional annotation and GSEA demonstrated the relationships between target genes and OS.

**Results:**

In this study, we discovered four key genes—*ALOX5AP*, *HLA*-*DMB*, *HLA*-*DRA* and *SPINT2* as new prognostic markers and confirmed their relationship with OS metastasis in the validation set.

**Conclusions:**

In conclusion, *ALOX5AP*, *HLA*-*DMB*, *HLA*-*DRA* and *SPINT2* were identified by bioinformatics analysis as possible prognostic markers for OS metastasis.

## Background

Osteosarcoma (OS) is the most common type of cancer that arises in bones and most people diagnosed with OS are under the age of 25 [1]. The incidence of OS in the general population is 2–3/million/year and the peak at the age of 15–19 is 8–11/million/year [2]. OS is characterized by early metastasis, poor prognosis without treatment [3], and more than 90% of patients die from lung metastasis before multiple chemotherapy. OS is currently undergoing multidisciplinary treatment, with approximately 15–20% of patients showing signs of metastasis at diagnosis, most in the lungs. Metastasis remains the leading cause of death in patients with OS, compared with 70% of patients with localized disease, and only about 20% becoming long-term survivors.

Previous studies have investigated mutational alterations or gene factors in an attempt to identify candidate OS driver oncogenes or tumors suppressors [4–6]. So far, for patients with metastatic OS, neither prognostic factors nor optimal treatment methods have been well established. Therefore, more attention must be paid to more precise risk assessment, not only for patient consultation, but also for determining treatment options based on reliable stratified criteria. In order to detect pulmonary metastasis OS early and improve poor survivorship, it is important to further explore more effective prognostic biomarkers and therapeutic targets.

Although research on biomarkers for metastasis within OS has recently expanded [7, 8], the targets after any OS diagnosis within the clinic and suitable for various sequencing platforms remain sparse. Recent development of gene chips and high-throughput sequencing technology, have enabled the identification of key genes related to tumor progression and prognosis based on big data integration and bioinformatics. Weighted gene co-expression network analysis (WGCNA) is a systematic biological method that could identify highly synergistically altered gene sets and screen out therapeutic targets or candidate biomarkers based on the inherent characteristics of the gene sets and the correlation between gene sets and phenotypes.

**Fig. 1:**
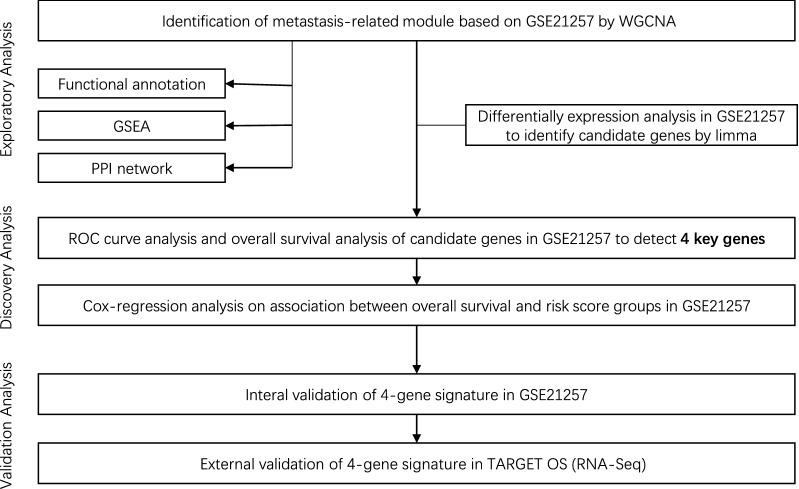
Flow chart of study design

Aiming at identifying and validating key genes in OS metastasis, the present study firstly identified associated module by WGCNA according to the gene expression profiles from Gene Expression Omnibus (GEO) datasets and determined the differentially expressed genes between metastatic OS samples and non-metastatic samples. Subsequently, Gene Ontology (GO) and Kyoto Encyclopedia of Genes and Genomes (KEGG) pathway analyses were performed to determine the most significant pathways associated with OS metastasis. Additionally, we constructed Kaplan–Meier (KM) curves and receiver operating characteristic (ROC) curves and screened the key genes related to OS prognosis. Moreover, univariate and multivariate Cox regression analysis were conducted to evaluate the predictive effect of the gene signature. Finally, we validated the gene signature using an external RNA sequencing (RNA-Seq) expression data obtained from The Therapeutically Applicable Research to Generate Effective Treatments (TARGET). The results may reveal the prognostic value of the gene signature for OS (Fig. 1).

## Methods

### Data sources and data preprocessing

We downloaded standardized matrix profile (*series matrix.txt) of GSE21257 (a microarray dataset) and obtained patient information from GEO database (Table 1) [9]. The platform of the dataset is the GPL10295 Illumina human-6 v2.0 expression beadchip. We removed probes not mapping to the Gene symbol using platform annotation file. For different probes corresponding to the same gene, their median expression values were taken as the final gene expression value. Differentially expressed genes (DEGs) between OS samples with metastasis and those without metastasis were identified using the “limma” (linear models for microarray data) R package (False discovery rate (FDR) < 0.05 and absolute of log2fold change (FC) > 1) [10].

**Table 1:** Clinical features of patients in the training set and validation set

	Training set		p	Validation set		p
	Metastasis	Non-metastasis		Metastasis	Non-metastasis	
Age			0.41			0.56
Median	16	18		14.05	14.37	
Gender			0.11			0.25
Female	9	10		12	25	
Male	25	9		9	38	
Grade			0.33	–	–	–
1	11	2				
2	9	7				
3	7	6				
4	3	2				

The OS RNA-seq expression data and the corresponding clinical follow-up data were obtained from the publicly available website of the National Cancer Institute TARGET Data Matrix (https://ocg.cancer.gov/programs/target/data-matrix). To meet the requirement for data analysis, we excluded the samples with incomplete information, then 84 OS expression data were remained. Genes that have average expression (Transcripts Per Million (TPM)   >  1) between samples were deemed as expressed. The expression value was processed as log2 (TPM + 1) for subsequent analysis.

### Constructing dynamic weighted gene co-expression network

We chose the 3000 most-varying genes for network construction and module detection. Specifically, the median absolute deviation (MAD) was used as a robust measure of variability. The network was built based on the protocols of R package WGCNA [11, 12]. We firstly clustered the samples to detect outliers. It appeared there was one outlier and we removed it by hand (Additional file 1: Figure S1A). Use PickSoftThreshold function to select β = 7 (scale-free R2 = 0.89) to build an adjacency matrix to make our gene distribution conform to scale-free networks based on connectivity for training set (Additional file 1: Figure S1B, C). Next, we used a blockwiseModules function to build a gene co-expression network in one step and a dynamic tree-cutting algorithm detected the modules. The parameters used for blockwiseModules function in WGCNA included a minimum module size of 30, and the dendrogram cut height for module detection set to 0.25 to define modules of co-expressed probesets. Meanwhile we calculated module eigengene of each module by measuring the first principal component of a specific module, which represented the overall level of gene expression within this module. Then, according to the correlation between the clinical traits and the module eigengene and the p-value to mine the modules related to the traits, we selected the module with the highest Pearson correlation coefficient for metastasis into subsequent analysis. Finally, to find hub genes for a given module, gene significance (GS, the absolute value of the correlation between the gene and the trait) and module membership (MM, the correlation of the module eigengene and the gene expression profile) were evaluated. Based on criteria of MM > 0.8 and GS > 0.2, hub genes in the blue module were screened. Cytoscape version 3.7.2 was used for network visualization. The above analysis is implemented using the R package “WGCNA”.

### Functional annotation and gene set enrichment analysis (GSEA)

R package clusterProfiler was used to conduct GO Biological Process (BP) [13] and KEGG biological pathway over representation analysis for interesting module genes [14]. GO terms and KEGG pathways with adjust p < 0.05 were considered statistically significant pathways. The enrichment analysis was implemented in command line of GSEA [15, 16]. An expression dataset and phenotype labels in the GSE21257 dataset were used to conduct GSEA analysis according to metastasis status (metastasis vs. non-metastasis). The data was then interrogated against Reactome gene sets (1499 gene-sets) from The Molecular Signatures Database (MSigDB) version 6.2 [17, 18]. We set the cut-off criteria as gene set size > 15, Number of enriched gene sets that are significant, as indicated by a FDR of less than 25%.

### Cox-regression based survival analysis

Univariate cox regression analysis was firstly performed to screen survival related genes. Furthermore, ROC analysis was performed to evaluate the predicting efficiency of the gene risk score and the area under curve (AUC) was calculated. The genes with p-value < 0.05 as well as AUC > 0.85 were screened as candidate genes for next analysis. These candidate genes were further selected for predictive signature construction. Risk scores were calculated and included in multivariate regression analysis in a Cox proportional hazard regression model for survival analysis. The Kaplan–Meier curve was used to visualize the survival probability for each group and p-value was calculated by the log-rank test. The survival analysis was implemented with package survival and survminer. The ROC analysis was performed using pROC package.

### Statistical analysis

Our study used a Wilcoxon rank sum test to compare continuous data between two groups. a Chi square test or Fisher’s exact test to test the difference between categorical variables. A p-value < 0.05 or a adjusted p-value < 0.05 was considered statistically significant. The Kaplan–Meier method and log-rank test was used to evaluate the correlation between gene expression and overall survival. The WGCNA method was analyzed by Pearson correlation analysis. All of these processes were conducted by R software (version 3.5.1 (×64)).

## Results

### Identification of key modules associated with OS metastasis

**Fig. 2:**
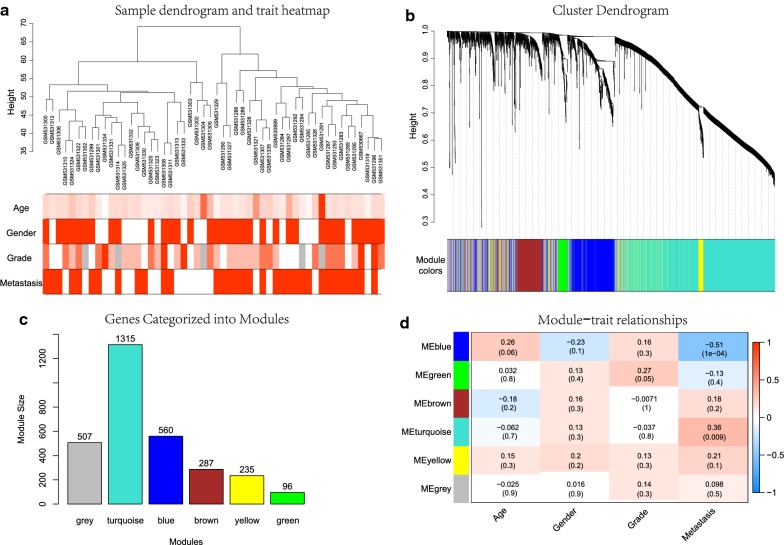
Construction and identification of modules associated with the clinical traits. **a** Clustering dendrogram of OS samples and the clinical traits. For age and grade, white means a low value, red a high value, and grey a missing entry; for gender and metastasis, white means female or non-metastasis, red means male or metastasis. **b** Hierarchical clustering based on the dynamic tree, each branch above represented a gene, and each color below represented a gene co-expression module. Grey module color is a reserved one for genes that are not part of any module. **c** Number of genes in different gene co-expression modules. Note that genes in the grey module were identified as not co-expressed. **d** Heatmap of the correlation between module eigengenes and clinical traits. Each row corresponds to a module eigengene, column to a trait. Each cell contains the corresponding correlation and p-value. The table is color-coded by correlation according to the color legend. The blue module was significantly correlated with metastasis

After data preprocessing and quality evaluation, an expression matrix with 3000 most varying genes and 52 OS samples with clinical information in GSE21257 was used for gene co-expression network construction (Fig. 2a). After merging similar modules, we were able to identify a total of six modules and each module was designated by distinct colors to distinguish between modules (Fig. 2b). The number of genes in each module were presented in Fig. 2c. Genes in grey module were removed in the further analysis. Additional file 1: Figure S1D allows us to visualize the interaction relationship of 5 modules. The representation showed a high-scale independence degree between any two modules even between genes within each gene module. Furthermore, eigengenes of all modules were calculated and clustered based on their correlation. The plot can be found in Additional file 1: Figure S1E, F. It is clear from this plot that the 5 modules were mainly divided into two clusters, which were consistent with the result of eigengene network heatmap. Next, relevance of all with all traits were assessed and results were presented in Fig. 2d. The highest correlation observed was for the blue module with metastasis (correlation coefficient values, − 0.51; p-values, 1e−04). In addition, the turquoise module was also found to be significantly related to metastasis (correlation coefficient values, 0.36; p-values, 0.009). Overall, we focused on the 560 genes in the blue modules in subsequent analysis.

### Functional annotation and GSEA

**Fig. 3:**
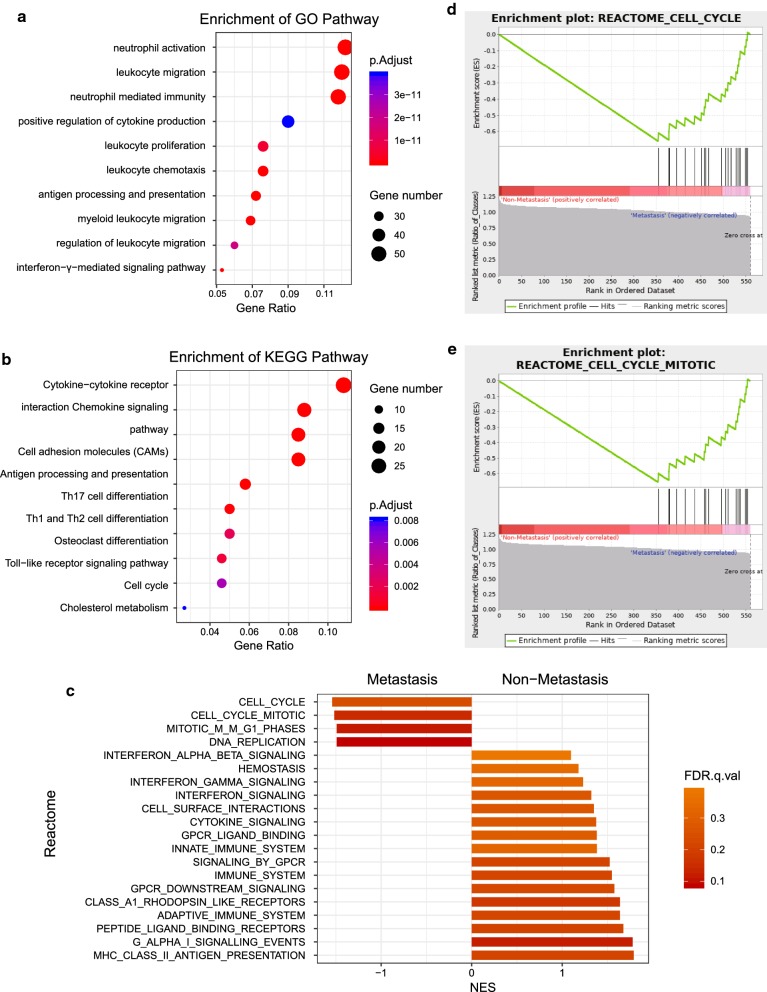
Functional enrichment analysis of blue module. **a** GO analysis of all genes in blue module. **b** KEGG pathway analysis of all genes in blue module. **c** GSEA in Reactome gene sets. **d, e** enrichment plots for cell cycle (**d**) and cell cycle mitotic gene set (**e**)

We conducted GO function and KEGG pathway analyses to examine the potential functional significance of the genes within blue module. BP of GO analysis showed that blue module was mainly enriched with cell migration, cell proliferation, cell cycle and immune response related pathway (Fig. 3a). Figure 3b presented the top 10 statistically significant observations of KEGG. The significant pathways included cytokine–cytokine receptor interaction, chemokine signaling pathway, toll-like receptor signaling pathway, cell differentiation, antigen processing and presentation and metabolism related pathway. In order to further understand the biological function of genes in blue module, GSEA was utilized to perform a pathway enrichment analysis and find enrichment of pathways defined by Reactome. Then we found 2 gene sets (cell cycle and cell cycle mitotic) were significantly upregulated in phenotype Metastasis in Reactome gene sets (Fig. 3c). The detailed results are available in Fig. 3d, e.

### Detection of hub genes based on the training set

**Fig. 4:**
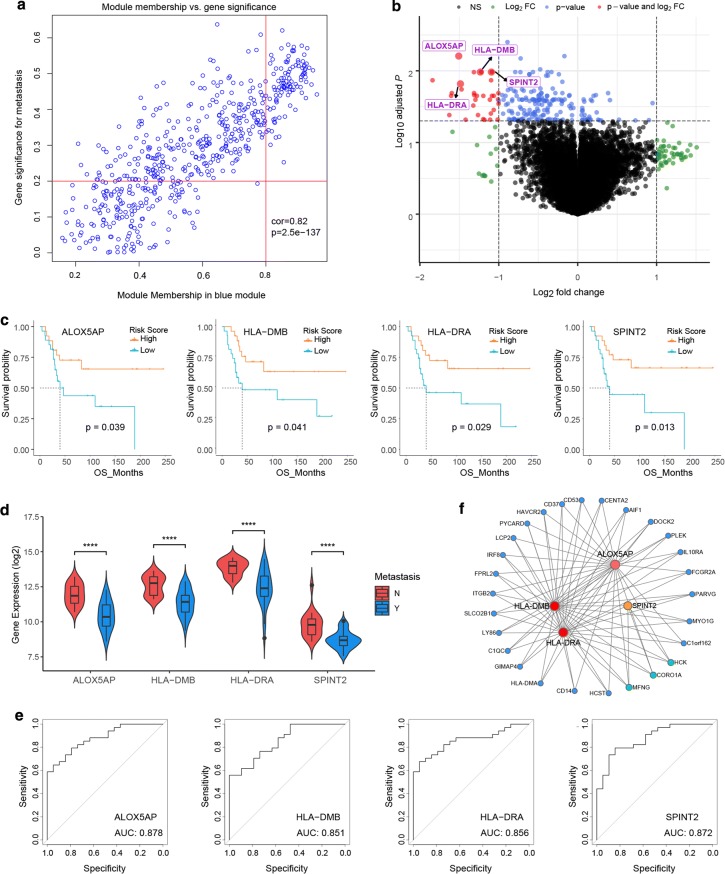
Identification of key genes based on training set. **a** A scatterplot of Gene Significance (GS) for weight vs. Module Membership (MM) in the blue module. There is a highly significant correlation between GS and MM in this module. **b** Volcano plot of significance of gene expression difference between metastasis and non-Metastasis patients. A gene is considered significantly differentially expressed if its |log(FC)| > 1 and p-value < 0.05. **c** Overall survival analysis of 4 key genes. Expression levels of ALOX5AP, HLA-DMB, HLA-DRA and SPINT2 are significantly related to the overall survival of patients with OS (P < 0.05). **d** Boxplot of significance of gene expression levels of 4 key genes. ALOX5AP, HLA-DMB, HLA-DRA and SPINT2 are significantly downregulated in metastasis OS compared with non-metastasis OS. The **** represents P < 0.0001. **e** ROC curves analysis of 4 key genes diagnosis. ROC curves and AUC statistics are used to evaluate the capacity to discriminate OS with or without metastasis with excellent specificity and sensitivity. **f** The network illustrates the relationship of 4 key genes and the 36 most frequently altered neighbor genes. The 4 key genes are presented in red and orange depending on the gene importance defined as the degree of connectivity. The other genes are represented in blue and green

As described in “Methods”, we analyzed the blue module and plotted MM against GS in Fig. 4a. All the DEGs were showed in Fig. 4b. After overlapping genes found by WGCNA and DEGs, we obtained 29 genes recognized as candidate genes. Among them, arachidonate 5-lipoxygenase activating protein (*ALOX5AP*), major histocompatibility complex, class II, DM beta (*HLA*-*DMB*), major histocompatibility complex, class II, DR alpha (*HLA*-*DRA*) and serine peptidase inhibitor, Kunitz type 2 (*SPINT2*) were negatively associated with the overall survival of OS patients (Figs. 4c). Moreover, the expression levels of these 4 genes were significantly higher in OS patients with metastasis, compared with non-metastasis patients (Fig. 4d). In addition, the diagnostic performance of these 4 genes was evaluated by ROC curves. The AUC showed that *ALOX5AP*, *HLA*-*DMB*, *HLA*-*DRA* and *SPINT2* indicated excellent diagnostic efficiency for patients with metastasis and those with non-metastasis (Fig. 4e). Figure 4f showed that *ALOX5AP*, *HLA*-*DMB*, *HLA*-*DRA* and *SPINT2* were highly connected in the network and demonstrated that the 4 genes play an important role in the development of OS.

### Evaluation and validation of 4-gene signature for survival prediction

**Fig. 5:**
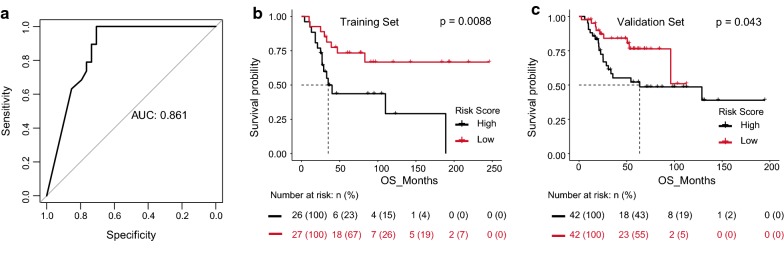
Evaluation and validation of the 4-gene signature risk model of OS. **a** The ROC curves are shown for risk score model in training set. **b**, **c** Kaplan–Meier analysis for the overall survival of OS patients in training set (**b**) and validation set (**c**)

To investigate whether the 4-gene signature could provide an accurate prediction of overall survival in OS patients, the 4-gene signature risk score were calculated for each patient in the training set according to the expression of these 4 genes for OS prediction. Then patients were divided into high- and low-risk groups using the median risk score as the cutoff. As expected, risk model might be a diagnostic marker for OS with an AUC of 0.861 (Fig. 5a) and patients with high-risk scores had a poor prognosis than those with low-risk scores (p = 0.0088) (Fig. 5b). As such, the 4-gene signature was validated using OS data from TARGET, and we achieved consistent results. KM curves revealed that the high-risk scores of 4-gene signature were significantly associated with shorter overall survival time of OS patients (p = 0.043) (Fig. 5c), which were similar to those observed in the training series. In order to further evaluate whether the expression levels of these four genes can provide good prognostic value, a multivariate Cox regression analysis was performed. The results can be seen in Table 2. It was evident that risk scores calculated from these four gene signature remained a strong independent prognostic factor for patients with OS (p = 0.02).

**Table 2:** Multivariate analysis adjusted for age, gender, grade, and risk score based on 4 genes signature in the training set

	HR	p
Risk score (low vs. high)	0.34	0.02
Age	1.00	0.88
Gender (male vs. female)	1.52	0.38
Grade (3 and 4 vs. unknown)	0.22	0.10
Grade (1 and 2 vs. unknown)	0.61	0.56

## Discussion

OS is the most common primary malignant tumor of bone, and the susceptible population is adolescents [19]. Its prognosis is very poor, and early metastases often occur. 20% of patients died of tumor metastasis or unresectable tumors, and the remaining 80% of patients had small metastases at the time of diagnosis. Many patients develop lung metastases within 1 year, and the 5-year survival rate is only 15%. Like many other malignancies, its etiology remains unknown [4]. Recently, a large number of new diagnostic techniques and effective chemotherapy methods have been developed, and the current 5-year survival rate has risen to 55–70%. Preoperative adjuvant chemotherapy followed by radical resection is still the most effective treatment. If surgical resection is not possible, radiotherapy may be beneficial for controlling local tumors [20]. As a generality, metastasis is the most adverse factors at diagnosis among known prognostic factors [21]. There are large differences in survival between patients with metastatic OS (10–20%) and non-metastatic OS (50–78%) [25, 26]. Moreover, metastatic OS are still very difficult to control and there are few effective therapeutic targets. Kinase targets, immune checkpoint inhibitors and cell surface marker GD2 have been actively investigated in multiple current clinical trials, but are inadequately evaluated [22, 23]. Therefore, further studies on early diagnosis or prediction of metastasis are warranted.

In our study, multiple bioinformatics analysis tools were used to identify 4 key genes related to metastasis and prognosis of OS patients, thus we constructed a risk score model which may benefit the treatment and prognosis evaluation of OS.

Using GO, KEGG and GSEA, we annotated the function of genes in the key module most related with metastasis, and clarified the underlying mechanism of metastasis in OS. Our results revealed that these genes were found to be enriched in cell cycle, cell proliferation, cell migration and immune response. Some researchers had demonstrated the functional link between cell cycle disorder and cancer cell invasion and metastasis [24, 25]. Several small pilot studies have reported that expression of molecules of tumor cell immune response, particularly HLA class II, can induce anti-tumor T cell responses, which may affect tumor progression and survival time of patients [26, 27]. Hence, we suggested that genes in blue module probably involved in the development and metastasis of OS through cell cycle pathway and immune response pathway.

After screening and filtering, we obtained four genes that may predict OS metastasis and have prognostic effects, and were evaluated in a cox regression model, indicating that it is an independent prognostic factor. The 4 key genes consist of *ALOX5AP*, *HLA*-*DMB*, *HLA*-*DRA* and *SPINT2*. *ALOX5AP*, also called 5-LO-activating protein (*FLAP*), which plays an important role in synthesis of leukotriene and associates with prognosis of primary neuroblastoma patients and esophageal squamous cell carcinoma patients [28]. Shi et al. [29] found that *ALOX5AP* showed strong associations in colorectal carcinoma due to microsatellite instability. And *ALOX5AP* has been considered as the important components of the leukotriene-synthesizing enzyme machinery, emerging opportunities for pharmacological intervention, and the development of new medicines exploiting both antiinflammatory and pro-resolving mechanisms [30]. Abelin et al. [31] revealed that *HLA*-*DMB* was dominated by professional antigen-presenting cells (APCs) rather than cancer cells. Aissani et al. [32] also found that antigen processing by *HLA*-*DMB* is a target pathway in the pathogenesis of HIV-related Kaposi’s sarcoma. Sun et al. [33] indicate that KSHV RTA facilitates evasion of the virus from the immune system through manipulation of *HLA*-*DRA*. Yokoyama et al. [34] demonstrate genetic overlap between AD and HLA-DRA and suggest that HLA-DRA influence AD pathogenesis and progression. *HLA*-*DMB*, one of the HLA class II beta chain paralogues, is expressed in antigen-presenting cells. Previous studies have confirmed that mRNA and protein levels of *HLA*-*DMB* are highly expressed in tumor samples from patients with advanced serous ovarian cancer with a large number of tumor-penetrating CD8 T lymphocytes, which can significantly prolong the median survival time [35]. *HLA*-*DRA*, a component of MHC II, alpha chain paralogues, also are expressed in antigen presenting cells. Both at transcription and protein levels, reduced expression of *HLA*-*DRA* has been shown to predict poor overall survival and progression-free survival in diffusive large B-cell lymphoma [36]. Moreover, enrichment analysis revealed up-regulation of immune response gene sets, including antigen presentation (*HLA*-*DMB* and *HLA*-*DRA*). What’s more, the *SPINT2* gene is epigenetically silenced or downregulated in human cancers, altering the balance of Hepatocyte growth factor activation/inhibition ratio, which contributes to cancer development and progression. Pereira et al. found that dysregulation of *SPINT2* is a common event in both pediatric and adult HGG, in which *SPINT2* may act as a tumor suppressor. *SPINT2* gene expression was down-regulated, altering dysregulation of the HGF/MET signaling pathway, which contributes to cancer development and progression [37, 38]. Whether these genes play the same role in the development and metastasis of OS deserves further study.

However, there were still some limitations in our work. Firstly, there are relatively small numbers of patients in two datasets obtained from publicly available database. In order to verify the stability and accuracy of the risk prediction model, more expression data and corresponding clinical information need to be collected, especially independent cohorts from multiple centers to further evaluate the applicability of the model. Secondly, our analysis is completely based on bioinformatics analysis, we need to accumulate more comprehensive experimental evidence, including in vivo and in vitro experiments. Finally, our analysis was entirely based on bioinformatics analysis to clarify the effect and possible molecular mechanisms of 4 genes on OS.

## Conclusions

In summary, we found 4 genes that may play a key role in OS metastasis and prognosis, and further constructed a risk score model, which may provide new clues for the prediction of OS metastasis and establish foundation to reveal prognostic markers and treatment targets for OS patients.

## Supplementary information

**Additional file 1: Figure S1**. Network construction and module detection. (A) Clustering dendrogram of samples based on their Euclidean distance. (B) Analysis of the scale-free fit index and mean connectivity for various soft-thresholding powers (β). Panels illustrate the scale-free fit index (y-axis) as a function of the soft-thresholding power (x-axis). Solid red horizontal lines are guides of the index at 0.9. At the power = 7, the index curve flattened out upon reaching the higher value in all groups. Effects of power values on the scale independence of genes co-expression modules for OS. (C) Effects of power values on the average connectivity of genes co-expression modules for OS. The panel displays the mean connectivity (degree, y-axis) as a function of the soft-thresholding power (x-axis). (D) Analysis of relationship between pairwise gene co-expression modules. Different colors of horizontal axis and vertical axis represent different modules. The brightness of yellow in the middle represents the degree of connectivity of different modules. There was no significant difference in interactions among different modules, indicating a high-scale independence degree among these modules. The modules in the horizontal and vertical axes were marked with different colors. The degree of the yellow brightness indicated the relevance. The overall relationship between the different modules was small, indicating that the modules had a high degree of independence. (E and F) The modules produced in the clustering analysis were summarized module eigengene dendrogram (E) and eigengene network heatmap (F). The eigengenes were mainly clustered into two clusters, containing 2 modules (modules green and blue) and 3 modules (modules brown, turquoise and yellow), respectively.

## Data Availability

The study data is available at GEO (https://www.ncbi.nlm.nih.gov/geo/query/acc.cgi?acc=GSE21257) and TARGET (https://ocg.cancer.gov/programs/target/data-matrix and choose “OS”).

## Abbreviations

OS: Osteosarcoma
GEO: Gene Expression Omnibus
TARGET: Therapeutically Applicable Research to Generate Effective Treatments
WGCNA: Weighted gene co-expression network analysis
GSEA: Gene Set Enrichment Analysis
ROC: Receiver operating characteristic
DEGs: Differentially expressed genes
FDR: False discovery rate
FC: Fold change
RNA-seq: RNA sequencing
MAD: Median absolute deviation
GS: Gene significance
MM: Module membership
GO: Gene Ontology
KEGG: Kyoto Encyclopedia of Genes and Genomes
AUC: Area under curve
*ALOX5AP*: Arachidonate 5-lipoxygenase activating protein
*HLA*-*DMB*: Major histocompatibility complex, class II, DM beta
*HLA*-*DRA*: Major histocompatibility complex, class II, DR alpha
*SPINT2*: Serine peptidase inhibitor, Kunitz type 2
