# Supplementary figures and images for: Expression profile analysis identifies key genes as prognostic markers for metastasis of osteosarcoma
Source: Cancer Cell Int. 2020 Mar 30;20:104. doi: 10.1186/s12935-020-01179-x (PMC7106759; doi:10.1186/s12935-020-01179-x)

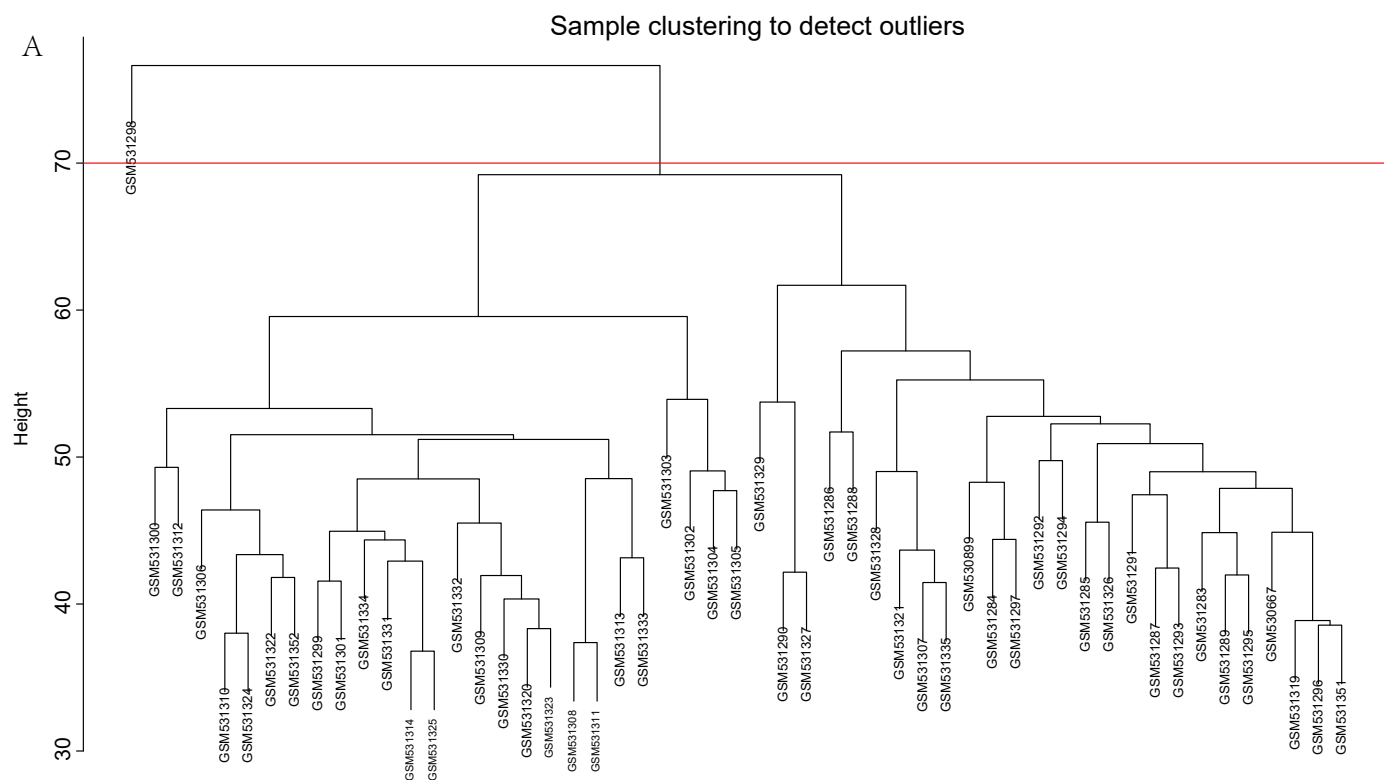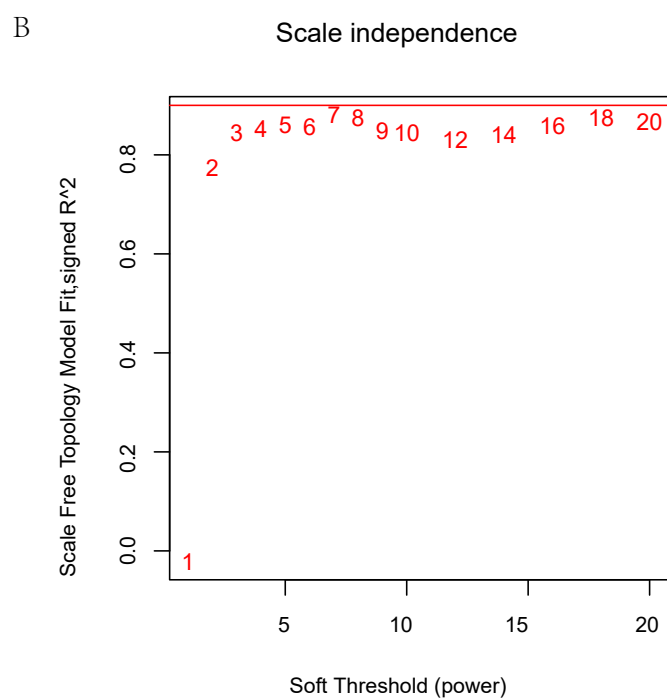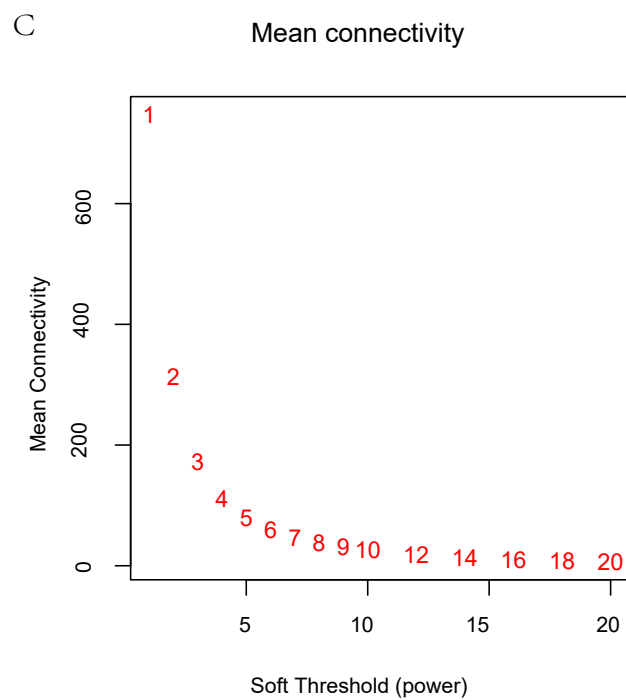

D

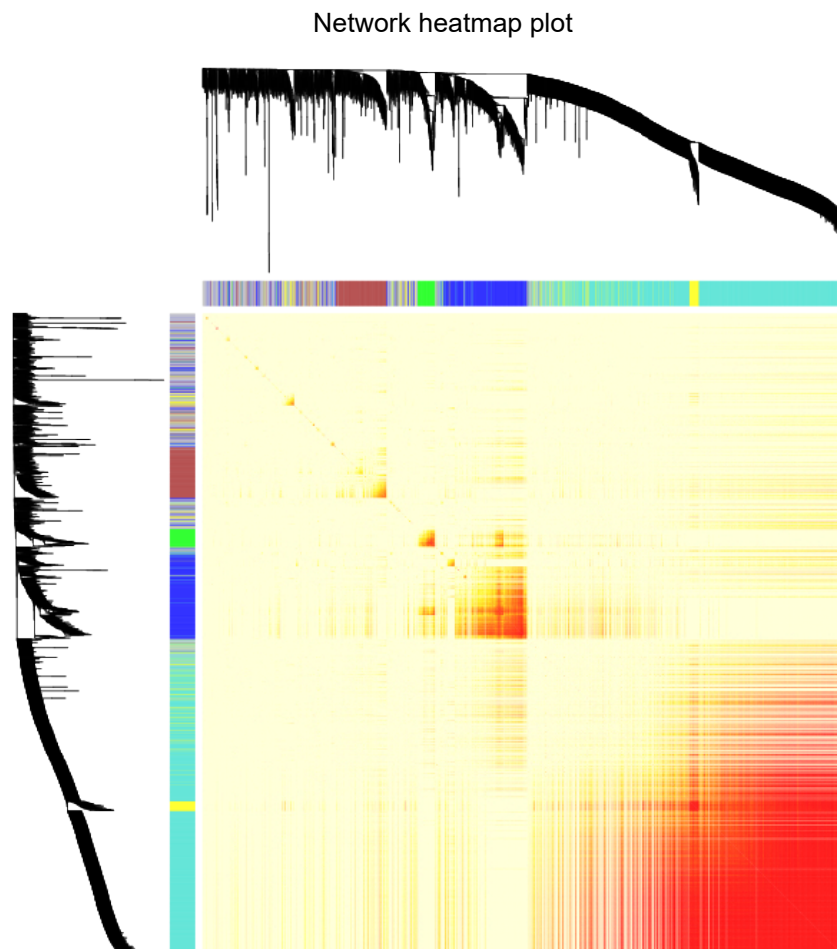

E

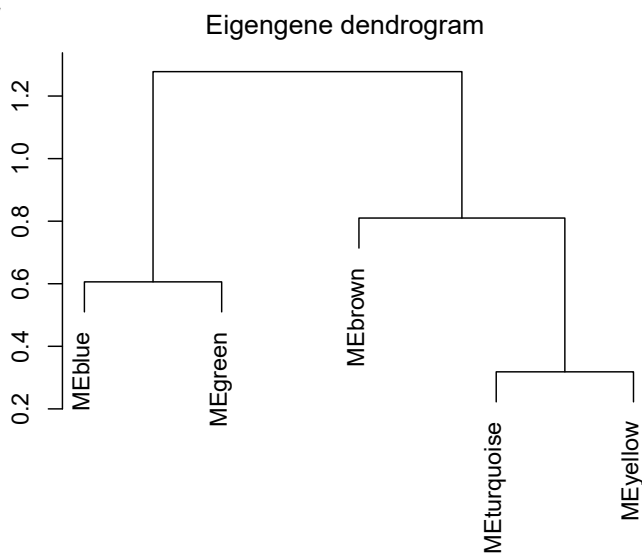

F

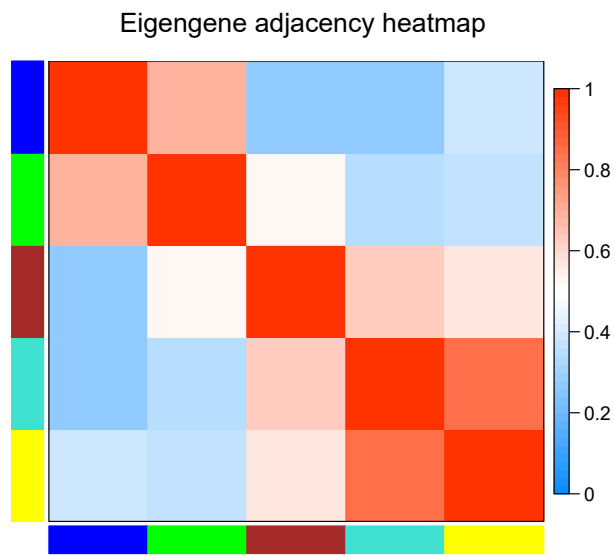

Supplement: Supplementary file 1 — Additional file 1: Figure S1. Network construction and module detection. (A) Clustering dendrogram of samples based on their Euclidean distance. (B) Analysis of the scale-free fit index and mean connectivity for various soft-thresholding powers (β). Panels illustrate the scale-free fit index (y-axis) as a function of the soft-thresholding power (x-axis). Solid red horizontal lines are guides of the index at 0.9. At the power = 7, the index curve flattened out upon reaching the higher value in all groups. Effects of power values on the scale independence of genes co-expression modules for OS. (C) Effects of power values on the average connectivity of genes co-expression modules for OS. The panel displays the mean connectivity (degree, y-axis) as a function of the soft-thresholding power (x-axis). (D) Analysis of relationship between pairwise gene co-expression modules. Different colors of horizontal axis and vertical axis represent different modules. The brightness of yellow in the middle represents the degree of connectivity of different modules. There was no significant difference in interactions among different modules, indicating a high-scale independence degree among these modules. The modules in the horizontal and vertical axes were marked with different colors. The degree of the yellow brightness indicated the relevance. The overall relationship between the different modules was small, indicating that the modules had a high degree of independence. (E and F) The modules produced in the clustering analysis were summarized module eigengene dendrogram (E) and eigengene network heatmap (F). The eigengenes were mainly clustered into two clusters, containing 2 modules (modules green and blue) and 3 modules (modules brown, turquoise and yellow), respectively. [file 12935_2020_1179_MOESM1_ESM.pdf]
